# HIV care experiences and health priorities during the first wave of COVID-19: clients’ perspectives – a qualitative study in Lusaka, Zambia

**DOI:** 10.1186/s12889-022-14493-y

**Published:** 2022-11-30

**Authors:** Njekwa Mukamba, Anjali Sharma, Chanda Mwamba, Herbert Nyirenda, Marksman Foloko, Kasapo Lumbo, Katerina Christopoulos, Sandra Simbeza, Kombatende Sikombe, Charles B. Holmes, Elvin H. Geng, Izukanji Sikazwe, Carolyn Bolton-Moore, Laura K. Beres

**Affiliations:** 1 Department of Research, Centre for Infectious Disease Research in Zambia, Lusaka, Zambia; 2 Department of Medicine, University of California San Francisco, San Francisco, CA USA; 3 London School of Hygiene and Tropical Medicine, London, UK; 4 Centre for Global Health and Quality, Georgetown University Medical Center, Washington, DC USA; 5 Division of Infectious Diseases, Washington University School of Medicine, St. Louis, MO USA; 6 Division of Social and Behavioural Interventions, Johns Hopkins Bloomberg School of Public Health, Baltimore, MD USA

**Keywords:** COVID-19, PLHIV, HIV care experience, Person-centred care, Zambia

## Abstract

**Background:**

The novel COVID-19 pandemic threatened to disrupt access to human immunodeficiency (HIV) treatment for persons living with HIV (PLHIV), two-thirds of whom live in sub-Saharan Africa. To inform a health system response supportive of continuity of care, we sought to understand clients’ HIV care experiences and health priorities during the first wave of COVID-19 outbreak in Lusaka, Zambia.

**Methods:**

Leveraging a study cohort of those who completed periodic SMS surveys on HIV care, we purposefully sampled 25 PLHIV after first confirmed COVID-19 case was reported in Zambia on 18^th^ March 2020. We phone-interviewed participants, iteratively refining interview guide to capture emergent themes on COVID-19 awareness, health facility interactions, and social circumstances, which we analyzed using matrix analysis.

**Results:**

All participants were aware of COVID-19, and HIV care experiences and health priorities of clients were affected by associated changes at health system, household, and individual level. The health system instituted early clinic visits to provide 6-months of antiretroviral therapy (ART) for stable patients and 3-months for unstable patients to reduce clinic visits and wait times. Most patients welcomed this long-desired extended appointment spacing. Some reported feeling respected and engaged when health care workers telephoned requesting their early clinic visit. However, others felt discouraged by an absence of physical distancing during their clinic visit due to ‘severe acute respiratory syndrome coronavirus 2’ (SARS-CoV-2) infection concerns. Several expressed a lack of clarity regarding next viral load monitoring date and means for receiving results. Patients suggested regular patient-facility communication by telephone and SMS. Patients emphasized that COVID-19 restrictions led to loss of employment and household income, exacerbating poverty and difficulties in taking ART. At individual level, most participants felt motivated to stay healthy during COVID-19 by ART adherence and regular laboratory monitoring.

**Conclusions:**

Clients’ HIV care and health priorities during the first wave of COVID-19 in Lusaka province were varied with a combination of positive and negative experiences that occurred especially at health system and individual levels, while at household level, the experiences were all negative. More research is needed to understand how patients practice resiliency in the widespread context of socio-economic instability. Governments and patients must work together to find local, health systems solutions to support ART adherence and monitoring. Additionally, the health system should consider how to build on changes for long-term HIV management and service delivery.

**Supplementary Information:**

The online version contains supplementary material available at 10.1186/s12889-022-14493-y.

## Background

COVID-19 and the restrictions associated with it had the potential to disrupt the health systems supporting HIV care [1]. Understanding the patient experience of the health system and social response to the emergency is necessary to ensure continued patient engagement in chronic disease care and support optimal health outcomes. While we are in a different phase of the COVID-19 pandemic, learning from context offers the opportunity to build more responsive and resilient health systems in the long run.

Extant literature shows that COVID-19 affected health systems in various ways including, disruptions of critical health services [2], reduced HIV testing [3], staff shortages, poor access to ART medication and delayed treatment as health care workers focused on treating patients with COVID-19 [4]. At the same time, individuals living with HIV had increased risk of death from COVID-19 infection [5]. Meagre evidence on the diverse effects due to COVID-19 at individual and health systems level [6] suggests that PLHIV maybe more susceptible to poor COVID-19 related outcomes and disrupted access to ART for the foreseeable future [7, 8]. A qualitative study conducted among women living with HIV in rural and peri-urban settings in Uganda showed that COVID-19 negatively affected re-engagement and retention in HIV care [9] Also, the health system had to re-adjust and re-allocate resources like staff and diagnostic equipment in a bid to combat the COVID-19 pandemic [10, 11]. In some sub-Saharan African countries, shortage of health personnel as a result of caring for COVID-19 patients led to suspension of other critical health services apart from ART drug dispensation [12].

To curb the spread of COVID-19 in Zambia, the government on 21st February 2020 announced COVID-19 screening at port of entry and 14-days self-quarantine for all in-coming travellers [13]. However, the Ministry of Health (MoH) reported the first two [2] cases of COVID-19 in Zambia on 18th March 2020 [14] and henceforth, the government strengthened public health measures aimed at preventing and mitigating the further spread of COVID-19 [13]. The public health measures which were enacted included restrictions on public and social gatherings, promoting positive health behaviours such as handwashing with soap, use of alcohol-based sanitizers, correct and consistent wearing of face masks and observing physical distancing of 1 to 2 m [15]. Both COVID-19 and these measures have brought about effects in health and livelihoods of people, but also with some negative consequences [16]. Additionally, community adherence groups (CAGs) became less attractive to patients following the introduction of COVID-19 measures in Zambia [17]. Prior to COVID-19 outbreak in Zambia, six multi-month dispensing (6MMD) was implemented as part of differentiated service delivery (DSD) model in which stable patients who were in care for more than 1 year with suppressed viral load were eligible for 6MMD and unstable patients would be given drug refills for 3 months or less depending on adherence history or viral load (VL) results. However, unique to the sub-Saharan region, the Zambian MoH accelerated the roll-out of 6MMD in which all stable patients were eligible for 6MMD and unstable ART patients defined as those who were viremic, lived with co-morbidities and aged between two to ten years were eligible for at least three multi-month dispensing (3MMD) [17, 18]. Additionally, the Zambian MoH also introduced and implemented an innovative approach of phone-calling patients back to the clinic for extended refills even before their scheduled clinic appointments [18].

However, understanding the client experience of HIV care and health priorities in Zambia, especially in the context of early visits and extended ART dispensation is lacking. Additionally, there is limited qualitative evidence about changes in health services access, patient care experience at health facilities, household dynamics, and patient expectations, all of which influence access and adherence to HIV care [19].

Given the uncertain and context-specific impact of COVID-19, we sought to understand clients’ HIV care experiences and health priorities during the first wave of COVID-19 outbreak, with the first reported cases on 18 March 2020 in Lusaka, Zambia. This will inform an ongoing health system response that is supportive of continuity of HIV care and offer lessons for resilience in the face of future public health emergencies.

## Methods

### Study setting

This qualitative study was nested within an on-going larger hybrid, implementation-effectiveness trial, *‘Person-Centred Public Health for HIV Treatment in Zambia’* (PCPH). PCPH was implemented across 24 Ministry of Health facilities in Lusaka Province, Zambia, supported by the Centre for Infectious Disease Research in Zambia (CIDRZ). The PCPH study was implemented from August 2019 to November 2021, with the aim of evaluating the feasibility, cost, cost-effectiveness, and effect of a multicomponent intervention intended to improve the patient-centeredness of HIV care. The PCPH intervention included: (1) training and clinic-based mentoring of health workers on patient-centeredness principles and skills, (2) systematic measurement of the HIV patient and health worker experience at the health facility using in-person and SMS-based exit surveys, and (3) motivating facility-level performance through provision of monetary and reputational incentives. Just over 6 months into the PCPH study, the COVID-19 pandemic interrupted routine study practices and service delivery in the health facilities. As part of the health facility measurements, we sought to understand patient experience and their ability to access and adhere to their ART regimen in order to support the Ministry of Health’s initiatives to ensure uninterrupted HIV care.

### Study population and sampling

Leveraging the existing PCPH cohort of adult patients (≥ 18 years) who provided periodic SMS survey feedback on their HIV care experiences, we purposefully sampled participants for variation in gender, preferred language (English, IciNyanja, IciBemba) and type of health facility where they sought care (Level 1 hospital versus health centers). ‘Level 1 hospitals’ refer to hospitals that have many health care workers with specialists providing specialized medical services while ‘health centers’ refer to clinics that have few health care workers and mainly focus on providing out-patient services [20].

### Study procedures and data collection

We collected data during Zambia’s first wave of COVID-19, between 29^th^ July and 17^th^ November 2020, approximately four months after the first confirmed COVID-19 case in Zambia. A team of three experienced qualitative researchers conducted formative, telephone-based, in-depth interviews in English, IciNyanja and IciBemba, using a semi-structured interview guide (See Additional file 1 for Interview Guide). Telephone-based interviews were utilized to limit COVID-19 exposure risk in the novel, pandemic environment.

We designed the interview guide to cover basic COVID-19 awareness, health facility interactions, and social circumstances, iteratively refining it to capture emergent themes (See Additional file 1 for final semi-structured interview guide). Additionally, we collected participants’ demographics that included age, gender, health facility size, duration of interview and socio-economic status (unemployed, self-employed, employed). To guide iteration, data collection was conducted in three rounds including with 10, 5 and 10 participants, respectively, per round, for a total of 25 interviews. After each round we conducted rapid analysis using a structured template and refined the interview guide to capture data on emergent themes in this novel context. We stopped data collection when saturation of themes was achieved. Prior to study implementation, the qualitative researchers were trained to gain familiarity with the study’s aims and fluency with the interview guide [21, 22]. Before data collection, participants gave verbal consent for participation and audio recording of the interview. One participant declined audio recording. Researchers took interview notes capturing key discussion points on all interviews. After data collection, approximately $6 was given to participants to ensure that there was no cost for their participation in the study. Researchers engaged in reflexivity exercise throughout the study. Memoing was done as soon as possible after conducting the interview and during analysis, debrief sessions provided an opportunity for researchers to identify their own positionality and improve self-awareness, in a bid to enhance credibility of study findings [23, 24].

### Data analysis

Interview notes together with all audio-recorded interviews (*n* = 24) were used to develop analytic memos [25]. The process of writing analytic memos was iterative. The data collectors wrote the initial draft of the analytic memo within 72 h of data collection to minimise recall biase. The structure of the analytic memo included, participants’ demographic information, facilitation context and responses under each of the interview guide questions. The initial draft of the analytic memo was reviewed by the first author who provided feedback on the content. The researchers who collected the data addressed the comments by listening to the audio-recordings and referring to the hand-written notes to ensure accuracy in notation and interpretation of the data. The senior researcher (AS) and senior author (LKB) also reviewed the analytic memos and asked clarifying questions to ensure data accuracy and completeness prior to finalization.

Matrix analysis was applied to finalized memos [26, 27]. The main deductive themes (matrix domains) were drawn from the interview guides and a summary excel-based template was developed. Four team members, trained in matrix analysis, used the excel-based template to jointly summarize data from one analytic memo to ensure that the domains were identifiable in the data and that there was consistency across team members in summarizing content under each domain. Once consistency was established, the four researchers summarised 6 analytic memos each using the template. Text from summary templates were then placed into each domain [28]. The process of summarizing or analysing the data included repeat reading of the analytic memos by the researchers and discussing the application of themes [29]. Any differences in interpretation of the data were resolved through dialogue among the researchers. Preliminary results were synthesized from the matrices by NM and reviewed by the senior researcher (AS).

### Ethical approval and consent to participate

Ethics approval was obtained for the study from University of Zambia Biomedical Research Ethics Committee. Verbal informed consent from all subjects was obtained and ethical approval was granted by the University of Zambia Biomedical Research Ethics Committee (UNZABREC), the University of Alabama at Birmingham (UAB). The National Health Research Authority and the Zambian Ministry of Health granted authority to conduct research. Prior to consenting, information about the study was given to the subjects using a written verbal consent script and thereafter, all subjects provided verbal consent before participating. All study methods were carried out in compliance with the Guidelines of Human Subject Protection and Declaration of Helsinki.

## Results

The study enrolled 25 participants and the median age was 43 years, with Interquartile Range (IQR) of 21 – 58, 52% were female (*n* = 13), and 64% were from health centers. The median duration of interviews was 56 min (IQR 24 – 113). Almost three-quarters (72%) of the participants were married and about one-third (36%) of the participants were unemployed. Participants’ awareness about COVID-19 was high (88%), based on ‘hearing about an illness called COVID-19’ and about three-quarters were knowledgeable about the existence of differentiated service delivery (DSD) models for HIV care such as Community Adherence Groups (CAGs), fast track and six multi-month dispensing (6MMD) (Table 1).

**Table 1:** Demographics characteristics of participants (*N* = 25)

	**N (%)**
Median age in years (IQR)^*^	43 (21 – 58)
**Gender**	
Male	12 (48)
Female	13 (52)
**Health Facility Type**	
Level 1 Hospitals	9 (36)
Health centers	16 (64)
**Median interview duration in minutes (IQR)**	56 (24 – 113)
Marital Status (Married)	18 (72)
Unemployed	9 (36)
Self-employed (Businessperson)	7 (28)
Employed	9 (36)
**Awareness about COVID-19**	22 (88)
Knowledge about Differentiated service delivery (DSD)^**^ Models	18 72)

^*^IQR Interquartile range is a measure of spread (or dispersion). It is defined as the difference between the upper and lower values or quartiles in a set of ordered data [30]

^**^ *DSD* Differentiated service delivery is a client-centered approach to HIV service delivery that aims to address client challenges whilst reducing the burden on the health systems [31]

We found that clients’ HIV experiences and health prioritises were influenced by the changes that occurred at the health systems, household, and individual levels due to COVID-19. The clients' experiences of care by health facility type were mainly the same across the three (3) themes of health system, household and individual level experiences. However, some differences include reduced client interaction with health care workers especially at hospital level. On the other hand, good communication of health care workers, bad attitude of health care workers, stigma, perceptions of COVID-19 prevention measures as a barrier to care, ART adherence and barriers to ART adherence due to drug side effects and food insecurity were shared by clients from health centers only (See Table 2 for results summary of client experiences by health facility type). In this section, we present the results along with some suggestions made by the clients.

**Table 2:** Summary of clients’ experiences of care by health facility type

No	Themes	Sub-Themes	Key Findings	Type of Health Facility	
				**Health Center**	**Level 1 Hospital**
**1**	**Health System Level Experiences**	a) Rapid scale-up of 6 multi-month dispensing (6MMD)	Consistent and longer drug refill was appreciated by clients	XX	XX
			Reduced clients’ waiting time due to decongestion of health facility	XX	XX
		b) Provision of high-quality health care services by health care workers	Clients appreciated good attitude of health care workers	XX	XX
			Clients appreciated good communication of health care workers	X	
		c) Complaints about service delivery	Bad attitude of health care workers	X	
			Reduced one-on-one interaction		X*
		d) Introduction of COVID-19 measures	General appreciation of COVID-19 prevention measures	XX	XX
			Perception of COVID-19 measures as barrier to care	X	
		e) Concerns	Fear of contracting COVID-19 infection	XX	XX
			Uncertainty about mechanism and timing of receipt of cluster of differentiation 4 (CD4) and viral load (VL) results	XX	XX
			Fear of HIV status disclosure/Stigma	X	
**2**	**Household Level Experiences**	a) Financial impacts	Loss of income	XX	XX
			Loss of employment	XX	XX
			Food insecurity	XX	XX
		b) Non-financial impacts	Disruptions in social interactions due to COVID-19 restrictions on movement and gatherings	XX	XX
**3**	**Individual Level Experiences**	a) Healthy behaviours	Motivation to stay healthy	XX	XX
			Motivation to religiously take ART medication	X	
		b) Unhealthy behaviours	Discouragement to take ART medication due to side effects and food insecurity	X	
		c) Laboratory Monitoring	Increased value for CD 4 and VL monitoring	XX	XX

**Key**

**XX** = Health Center & Level 1 Hospital; **X** = Health Center only; X* = Level 1 Hospital only

### Health system level experiences

Clients reported health system changes that included (a) rapid scale-up of 6 multi-month dispensing (6MMD), resulting in reduced waiting time and congestion at health facilities during of ART pick-up, (b) provision of high-quality health care services by health care workers (HCWs), (c) complaints about service delivery and (d) introduction of COVID-19 prevention measures.

#### Rapid scale-up of 6MMD

Clients described rapid scale-up of 6MMD across most health facilities across Lusaka province with a male patient from a health center specifying that “Since COVID 19 started, the health facility now gives patients medication for a six-month period”. Clients reported receiving phone calls from health care workers encouraging them to make early visits to the health facility for drug pick-up. They recommended consistent and longer drug refills for clients beyond the COVID-19 era with a male client from a Level 1 hospital, suggesting that “Dispensing of medication for longer periods of time is a good thing compared to times when we were in CAGs,” in reference to the prevailing differentiated care models during pre-COVID-19 times.

According to the clients, the rapid scale-up of 6MMD where stable clients received 6-months of ART drug supply and unstable clients received 3-months refill, resulted in reduced waiting time and decongestion of health facilities such that “after COVID 19, [clients] spend less time at the health facility [being] attended to within 30 min.” Some clients recognized that the health facilities had strategized “to decongest the clinic by fast tracking stable ART clients. Health care workers only spend more time on clients that require medical diagnosis by medical personnel.” [Male, Health center, IciBemba].

#### Provision of high-quality health care services by health care workers (HCWs)

When asked about the changes related to HIV care and treatment due to COVID-19, clients opined that health care workers provided better quality health care services during this time. Clients felt happy and respected when receiving early visit phone calls by health care workers who took time to explain the reasons for the unexpected and early visit. Additionally, clients were pleased with the interaction they had with the health care workers, and expressed appreciation of the welcoming attitude, friendliness, respect, attentive listening, and shared decision making about next visit appointment date.

“I saw care from health care workers. I was among the clients that regularly received phone calls from health care workers inviting us to visit the health facility for drug pick-up. This clearly showed that the health care workers care for clients – I am happy with the way health care workers are now behaving – they now greet clients.” [Male, Level 1 hospital, IciNyanja].

“During COVID-19, the quality of HIV service I received from health care workers was good because I was welcomed in a friendly manner and was greeted in such a way that made me feel comfortable at the health facility.” [Female, Health center, English].

#### Complaints about service delivery

Conversely, some clients complained about the bad attitude of some health care workers which was linked to poor communication and reduced one-on-one interaction time. Poor communication included not explaining changes, for instance, details of drugs dispensed, and getting a rude response when asking about these changes. Clients took more umbrage to rudeness in the vernacular, where politeness is the norm. This rudeness can anger patients who may disengage from HIV care.

“I was given new drugs without an explanation or reason for change of drugs. When I asked why the health care worker had given me a different drug, they responded rudely in IciBemba, saying “ngamulefwaya mupose” [If you want, you can throw it away].” [Female, Health facility, IciBemba].

Additionally, clients noted that the shortened clinic visit affected the quality of care as they did not receive individual attention or get a listening ear from some healthcare workers:

“Now, they are not spending time on one-on-one interaction with us when we visit the hospital. Before, you could even tell the health care worker privately what is bothering you. But now they will quickly tell you, “Okay we are done, call the next patient!” And instantly I get discouraged, and I walk out.” [Male, Level 1 hospital, English].

#### Introduction of COVID-19 prevention measures

Most clients noted that “many changes as the result of COVID-19” were introduced at the health facility. Some of the COVID-19 prevention measures highlighted by clients included mandatory and proper wearing of face masks, observing physical distancing, use of alcohol-based hand sanitizer, and hand washing with water and soap at the entrance of health facilities. While generally appreciative of these precautions to prevent COVID transmission, some patients observed the enforcement of COVID-19 prevention measures as a barrier to care, “I have noticed clients being turned away from the health facility if they do not have face masks” [Male, Health facility, IciNyanja].

Notwithstanding the evidence of mostly favourable health system experiences highlighted above, clients raised some concerns about the COVID-19-related changes affecting HIV care. This included fear of contracting COVID-19 infection, uncertainty about mechanism and timing of receipt of cluster of differentiation 4 (CD4) and VL results and fear of HIV status disclosure. Clients were concerned about acquiring nosocomial COVID-19 infection, a fear triggered by observing some clients who did not observe physical distance. They also expressed anxiety about being exposed to COVID-19 when taking public transport to and from the health facility. The uncertain knowledge on modes of COVID-19 transmission exacerbated these fears “of getting infected with the COVID-19 virus because” as one female client from a Level 1 hospital explained, “I have heard that if you sit where an infected person sat before you, then you can get the COVID-19 virus.”

Some clients were unclear about their next viral load monitoring appointment date which worried them because as one female client explained, “it is what helps monitor my viral suppression”. Additionally, they were concerned that they would not receive their CD4 and viral load laboratory results, a common practice during pre-COVID-19 times in some health facilities. In a bid to improve communication, one male client from a health center suggested “I would prefer to be contacted via phone call, text or a health care worker can visit me at home to share any relevant information”.

Following the early visit phone calls made by health care workers, some clients were concerned with possibility of unintended status disclosure resulting in stigma, if they attended a clinic visit at a time when many other people were being called to do the same, thereby increasing the chance of meeting people whom they know at the health facility during the early visit “that can go and start telling people in the community” about their HIV status. Additionally, changes in drug dispensation, for example, from the pharmacy room to pharmacy window in some pharmacy departments, made some clients uncomfortable that their HIV status would be involuntarily disclosed.

“What had changed regarding HIV care was that clients used to enter the pharmacy room one by one to collect drugs before COVID-19. But currently, people are asked to line up outside and collect ART drugs from the window thereby exposing ART clients to unintended disclosure when they are spotted by people they know:” [Female, Health center, IciBemba].

### Households level experiences

Financial and non-financial impacts were the main experiences by clients at household level during the epidemic. Financial impacts included loss of income or salary cuts which were introduced by employers to mitigate against negative effects of COVID-19. Non-financial impacts included social and health losses as described below. Financial impacts were the prominent concerns raised by clients during the interviews.

#### Financial impacts

Clients emphasized that COVID-19 restrictions led to loss of employment and household income, thereby exacerbating food insecurity. Clients perceived that COVID-19 brought reduced household income due to loss or curtailing of business, jobs, and informal employment. In some cases, clients lacked transport money to go to the health facility for drug pick and this affected their adherence to ART.

“I have been financially impacted due to the loss of a job, and this has resulted in food insecurity in my home. When I take my HIV medication, the side effects become severe if I have not eaten and I end up experiencing drowsiness and body weakness.” [Female, Health center, English].

“My working conditions have changed. I work as a builder. But due to COVID-19, the company I work for instructed me to only report for work when they tell me to do so, and my salary has changed. I now only gets paid according to the number of days I have worked in a month. This has affected my supply of household necessities including handwashing soap for my family and food [to eat].” [Male, Level 1 hospital, IciNyanja].

In view of this, some clients suggested food support in the form of mealie meal and high energy protein supplements (HEPS) meant to help them to stay healthy and boost their immunity.

#### Non-financial impacts

COVID-19 restrictions imposed by the Government of the Republic of Zambia on peoples’ movements and social gatherings in schools and churches led to disruptions in social interactions as families were unable to freely visit friends and relatives. Disrupted social interactions due to COVID-19 restrictions on movement and gatherings led to health problems as clients lost their social support systems to help them cope with living with HIV.

“Children are not going to school. We are not going to Church and there are restricted movements. The impact of COVID-19 on people living with HIV is restricted movements and this does not allow for an active social life, which helps with coping with the HIV virus.”[Male, Level 1 hospital, IciNyanja]

### Individual level experiences

Individual level experiences caused by COVID-19 as narrated by patients included an increased perceived threat to their health that motivated them to adopt healthier behaviours and increased the value they placed on laboratory monitoring, but which was challenged by COVID-associated financial and livelihood threats.

#### Motivation to adopt healthier behaviours

Most clients felt their HIV status weakened their immunity and made them more prone to COVID-19 infection and to severe disease which could escalate to death. This threat posed by COVID-19 motivated them to stay healthy and preserve their lives during the COVID-19 epidemic by adopting healthy eating and exercise habits to boost their immune system and by religiously taking ART medication to suppress their viral load.

“I want to be healthier in this time of COVID-19 compared to before because I feel that having HIV puts me at a higher risk than a person who does not have HIV. I feel that I have a weaker immunity and medication must always be taken to ensure that I stay healthy and avoid getting COVID-19.” [Female, Health center, English].

Conversely, some clients felt discouraged taking their ART drugs during COVID-19 due to side effects which was worsened by food, nutrition, and income insecurity.

“Lack of sufficient food is the main reason that makes taking ART drugs hard because when the medication is taken on an empty stomach, I feel nauseous and weak until I find some food to eat, then I feel better.” [Female, Health center, IciNyanja].

#### Increased value for CD4 and viral load monitoring

The concern for their health and the desire to live increased the value of CD4 and viral load monitoring during the COVID-19 era among clients, moved them to desire information to make any changes necessary to stay healthy and avoid death.

“I view the labs for my CD4 and viral load as more important now than before COVID-19 so I can know the state of my health” [Female, Health center, IciNyanja].

For these reasons clients wanted improvements in communication about their laboratory test results with a client from a Level 1 hospital suggesting that they should know their results within 2 days and be prepared with this information for their next ART appointment:

“Ministry of Health in Zambia should introduce a laboratory system which will give results for viral load and CD4 count within 24 h or 48 h upon specimens’ collection. So that as cliients go for their next ART appointment visits, they are already aware of how they are faring with regards to lab monitoring. Lab monitoring is the only way clients can have information about their immune system and viral suppression.” [Male, Level 1 hospital, English].

## Discussion

Clients experienced changes in their HIV care and health priorities during the first wave of COVID-19 in Lusaka, Zambia. Associated changes at health system, household, and individual levels differentially impacted the ability of clients to access and adhere to HIV care. Generally, we did not see any major differences in clients’ experiences and services received by health facility type, except for reduced patient-provider interaction that was shared only by clients from Level 1 hospital. The reduced patient-provider interaction was mainly due to the COVID-19 protocols which were enforced in a bid to prevent further infections. These results present the Zambian context and are also consistent with a study conducted in South Africa that showed that clients were reluctant to visit hospitals during COVID-19 era for fear of exposure [32].

At health system level, the rapid scale up of 6MMD across health facilities not only increased access to ART drugs but also satisfied clients since health facilities became de-congested and reduced clients’ waiting time. Some clients applauded HCWs for providing person-centred and quality HIV care services. Nonetheless, clients raised concerns regarding rudeness from some HCWs, possible COVID-19 infections, potential unavailability of CD4 and VL results and unintended status disclosure resulting in stigma. At household level, clients experienced socio-economic changes that took the form of reduced income and food security, which along with social distancing, reduced their ability to cope with HIV and ART side-effects. At individual level, clients strove to stay healthy by adhering to ART treatment and CD4 and VL monitoring to avoid bad health outcomes.

The COVID-19 pandemic motivated clients to stay in HIV care for fear of contracting it and having worse health outcomes. This could be attributed to the good knowledge (88%) about COVID-19 that clients demonstrated. These results build on existing evidence in sub-Saharan Africa, particularly a study conducted in Ethiopia which showed that all participants (100%) responded correctly to all preventive knowledge questions [33]. In contrast, a study conducted in Cameroon revealed that 21.9% of study participants had correct knowledge of COVID-19 [34] and older population and people with underlying health conditions were at highest risk of COVID-19 [34].

Our study shows that clients especially in health centers consistently took their ART medication at the prescribed time benefiting from the rapid scale-up of 6MMD that occurred in Zambia to counter the stringent restrictions of the first COVID-19 wave. Interruptions to ART drug supply have potential to lead to death of clients [35]. Taking of ART medication is therefore linked to access of drugs and subsequent good health outcomes of clients. While our study confirm that clients adhered to their ART medication, a study conducted in South Africa, found that one-fifth of HIV-infected patients were unable to get antiretroviral medications refills due to the COVID-19 pandemic [36]. Consistent with our findings, other PEPFAR-supported countries in sub-Saharan Africa also increased ART dispensation from three to six months in order to de-congest health facilities and limit transmission of COVID-19 [37]. Other strategies to improve access during periods of restricted movement include decentralising ART drug supply using other differentiated service delivery (DSD) models such as home deliveries and community adherence groups [38]. Such strategies can also reduce stress on the health care system, which must deal with managing the influx of COVID-19 patients. Finally, national governments should ensure that supply chain of medical products is strengthened, and adequate ART drug stocks are available in all health facilities in order to have sustained provision of ART drugs to clients.

Our data suggest that the health system adjusted to ensure that clients were served efficiently during clinic visits and instituted measures such as early visit invitations made over phone calls, enforcement of COVID-19 prevention guidelines and de-congestion of clinics. Similarly, a study conducted in Namibia highlights clinic adjustments such as screening and triaging of clients for COVID-19 symptoms and limiting clinic appointments to avoid over-crowding [39]. These public health measures were part of adaptation measures in the context of COVID-19 so that clients were in sustained HIV care. Additionally, these measures assured clients of governments’ concern for their safety and well-being and prevent further spread of COVID-19 among clients and HCWs, which can lead to further disruption of HIV and other routine service delivery [40].

Our findings contribute a clearer understanding of the socio-economic impact of COVID-19 among clients, resulting in unemployment, food and income insecurity at household level. These results build on existing evidence thatCOVID-19 measures such as partial lockdowns, travel bans, closing of schools, companies and large gathering bans had adverse effects on economies in sub–Saharan Africa [41]. In such circumstances, scarce resources and employment seeking may be directed towards survival and family welfare rather than continuity in HIV care and consequently, the public health measures were seen to indirectly threaten continuity in HIV care for some clients. For example, a client without regular food intake has to choose between taking ART medication on an empty stomach at the risk of adverse side effects or to discontinue taking the drugs. In contrast, the African Cohort Study conducted in Tanzania, Uganda, Kenya and Nigeria found that COVID-19 had no negative effect on ART adherence [42]. This was on account of the Community-based HIV care which provided good social support system that fostered adherence. Therefore, in order to mitigate against adverse effects of pandemics on clients that live in similar contexts as Zambia, food security and nutritional assessments should be integrated as part of screening and triaging process during clinic appointment visits for clients [43]. Additionally, in times of pandemics like COVID-19, national governments should strengthen social protection systems that respond to the needs of vulnerable populations like clients [31].

The COVID-19 and other infectious pandemics can create fear of being infected with COVID-19 while being HIV positive as reported by us and a qualitative study conducted in Rakai region of Uganda [44]. In our study, this fear led some clients to seek viral load and CD4 monitoring in order to stay healthy and avoid bad health outcomes. Conversely, some clients feared that their HIV status would be disclosed if they met friends or relatives at the health facility for drug pick-up. These results build on existing evidence of a Lusaka-based study that found that fear of COVID-19 infection and anticipated stigma affects health seeking behaviour at a time when clients are most vulnerable [45]. Unintended HIV status disclosure resulting in stigma and fear along with social isolation have implications for mental health, which may already be a co-morbid condition among clients [46, 47]. Therefore, interventions aimed at reducing fear, stigma and promoting mental health and wellbeing among clients should be prioritised and integrated in the existing interventions for clients especially during pandemics like COVID-19. Additionally, there is need for more research to understand clients’ perceptions on acceptability and feasibility of virtual psychosocial support services using phone-based communication, during periods of pandemics like COVID-19.

There are some limitations to this study. Firstly, the interviews were conducted during the first wave of COVID-19 and given the rapidly evolving nature of this pandemic, the experiences of clients might have changed in subsequent waves. However, capturing the changes over time in the experience of clients is beyond the scope of this study. Secondly, due to the relatively small sample size of this qualitative research, the study participants may not be representative of all PLHIV in Lusaka province. Nevertheless, given that participants were drawn from 12 diverse health facilities in Lusaka and that we achieved thematic saturation, we are confident that these findings are representative of the experiences of clients during the first wave of COVID-19 in Zambia. Thirdly, given that interviews were conducted over the phone, interviewers were not able to observe participants’ non-verbal and contextual cues that would otherwise be present in a face-to-face interview. However, trained and experienced interviewers were able to listen for tone [48, 49] and apply interviewing and probing skills to minimize social desirability bias during data collection.

## Conclusions

Clients’ HIV care and health priorities during the first wave of COVID-19 in Lusaka province were varied with a combination of positive and negative experiences that occurred especially at health system and individual levels, while at household level, the experiences were all negative. Clients’ positive experiences included rapid scale up of 6 multi-month dispensing, high quality health care provided by health care workers, motivation to adhere to ART and increased value for CD4 and viral load monitoring. Additionally, clients shared negative experiences that included bad attitude of some health care workers, fear of nosocomial infections, stigma, loss of employment and income and food insecurity, all of which threatened their continuity in HIV care. 

Clients have benefitted from the long history of PEPFAR-supported ART programs which has increased trust and interaction with the health system in many countries, both of which kept clients in care even during COVID-19 era. Additionally, countries were already shifting to DSD models and so had the nimbleness both in the health system and in client cohorts to absorb and shift to 6MMD.

More research is needed to understand the HIV care experience and priorities among clients in the subsequent waves of COVID-19. Governments and other key stakeholders need localised interventions for clients that would mitigate the socio-economic, psychosocial, and health-related effects of COVID-19, thereby supporting improved HIV retention and viral load suppression. Additionally, deliberate efforts in addressing stigma concerns and social isolation among clients during pandemics should be prioritised by providing psychosocial and mental health support.

## Supplementary Information

**Additional file 1.**

## Data Availability

The transcripts and datasets generated and/or analysed during this qualitative study are not publicly available due to privacy provisions but are available from the corresponding author on approval from the authorizing institutional review board.

## Abbreviations

AIDS: Acquired immunodeficiency syndrome
ART: Antiretroviral therapy
CAGs: Community Adherence Groups
CD4: Cluster of differentiation 4
CIDRZ: Centre for Infectious Disease Research in Zambia
DSD: Differentiated service delivery
HCWs: Health care workers
HEPS: High energy protein supplements
HIV: Human Immunodeficiency
IQR: Interquartile Range
IRB: Institutional Review Board
MoH: Ministry of Health
PCPH: Person-Centred Public Health for HIV Treatment in Zambia
PEPFA: President’s Emergency Plan for AIDS Relief
PLHIV: Persons living with HIV
SARS-CoV-2: Severe acute respiratory syndrome coronavirus 2
6MMD: Six multi-month dispensing
3MMD: Three multi-month dispensing
UAB: University of Alabama at Birmingham
UNZABREC: University of Zambia Biomedical Research Ethics Committee
VL: Viral Load
