# Supplementary material for: HIV care experiences and health priorities during the first wave of COVID-19: clients’ perspectives – a qualitative study in Lusaka, Zambia
Source: BMC Public Health. 2022 Nov 30;22:2238. doi: 10.1186/s12889-022-14493-y (PMC9713144; doi:10.1186/s12889-022-14493-y)
Supplement: Supplementary file 1 — Additional file 1. [file 12889_2022_14493_MOESM1_ESM.docx]

**Appendix 1: Semi-structured Interview Guide – Changes arising from COVID-19**

| **Qualitative Formative Interviews on HIV Patient Experience and Patient Centredness in the Context of COVID-19** | |
| --- | --- |
| 1. What is your age? |  |
| 1. Have you heard about an illness called COVID-19/corona/coco? | ⭘ Yes  ⭘ No |
| **We would like to speak with you about living with HIV and accessing HIV care in the context of COVID-19/corona.** | |
| 3. What, if anything, has changed regarding your HIV care and treatment due to COVID-19? *(****What change has happened since the coming of covid-19) How has this affected your HIV care?***  {covering Penchansky’s: Availability, Accessibility, Acceptability, Accommodation, COVID domains of routines, employment/income, resource access, emotions/worries}  Probes:   - 1. Anything that makes **getting** ART harder? [scheduling, transport, fear of COVID-19, Govt directive, loss of job or income, change in household role, additional stress or worry, change in routines due to virus, difficulty getting food or other resources, no mask / hand sanitizer / gloves]   2. Anything that makes getting ART easier? Are you more free to come after having missed a visit because everyone gets 6 months of ART?   3. Anything that makes **taking** ART harder? [loss of job or income, change in household role, additional stress or worry, change in routines due to virus, difficulty getting food or other resources, moving household location]   4. Anything that makes taking ART easier?   5. Did you rely on community-based drug collection or other health support that is not available now?   6. Concern that you may not get routine labs such as CD4 or viral load – and you’d like to have that information?   information you would like to have.   - 1. Experience of going for a visit early and collecting a 6-month drug supply?   2. (If recent visit) How were your interactions with the health care workers?      1. Shared decision making?      2. Good communication: given good information? Listened to? |  |
| 4. What, if anything, do you think should change related to your HIV care and treatment due to COVID-19?  {covering Penchansky’s: Availability, Accessibility, Acceptability, Accommodation}  Probes   1. Anything you can think of that would make getting ART and taking ART easier / harder? Do you have any concerns about going to the facility? (How do you feel about going to the facility during this time?   What do you like about it?  What do you not like about it?   1. [note: we are interested if people may fear going to the facility because it could risk exposing them to COVID-19. However, we DO NOT want to introduce fear! So it is important to stay open-ended and see what comes up] 2. Concern about contracting COVID-19 at the facility? Concern about contracting COVID-19 when traveling to the facility? Concern about being turned away due to not wearing a mask? Other? 3. You could ask 'did you participate in a CAG or UAG or fast-track or scholars pick up' If they say 'yes', you could ask 'what do you think about that approach now that COVID-19 is here? 4. Due to COVID-19, many facilities are giving patients 6 months of ART. What do you think about that length of time? (Probe: too long? too short? what would be better?   **If the participant struggles to understand:**  (Maybe start with this?  How did you typically pick up your medicines, for example, this time last year? (I think most urban facilities its only MMS & FT)  How would you feel if you had to follow the same process now?  How many days/months of medicine do you think should be given to people who are generally doing well (i.e. have suppressed VL)  What process are you following now to pick up your medicines?) |  |
| 5. What, if anything, has changed in your life or household due to COVID-19?   - 1. loss of job or income, change in household role, additional stress or worry, change in routines due to virus, difficulty getting food or other resources, moving household location, specific COVID-19 prevention measures?   Follow-up: How has that impacted living with HIV? |  |
| 6. What information, if any, would you want from health care workers about HIV at this time? |  |
| 7. What sort of support to live healthy with HIV would you like at this time?   1. Who provides this support for you? 2. What do you need that you do not have? 3. How do you understand COVID-19 versus flu? |  |
| 8. What other health concerns do you have now?   - 1. How do you feel about going to clinic to take care of this concern? |  |
